# (Mis)alignment of employer support and needs of healthcare providers in end-of-life care during a healthcare crisis: a longitudinal mixed-method study during the COVID-19 pandemic (the CO-LIVE study)

**DOI:** 10.1177/26323524241308268

**Published:** 2025-10-08

**Authors:** Masha S. Zee, H. Roeline Pasman, Erica Witkamp, Yvonne N. Becqué, Anne Goossensen, Bregje D. Onwuteaka-Philipsen

**Affiliations:** Department of Public and Occupational Health, Expertise Center for Palliative Care, Amsterdam UMC, VU University, Van der Boechorststraat 7, Amsterdam 1081 BT, The Netherlands; Department of Public and Occupational Health, Expertise Center for Palliative Care, Amsterdam UMC, VU University, Amsterdam, The Netherlands; Department of Public Health, Erasmus MC University Medical Center, Rotterdam, The Netherlands; Research Center Innovations in Care, Rotterdam University of Applied Sciences, Rotterdam, The Netherlands; Research Center Innovations in Care, Rotterdam University of Applied Sciences, Rotterdam, The Netherlands; University of Humanistic Studies, Utrecht, The Netherlands; Department of Public and Occupational Health, Expertise Center for Palliative Care, Amsterdam UMC, VU University, Amsterdam, The Netherlands

**Keywords:** COVID-19, emotional support, employer support, healthcare providers

## Abstract

**Background:**

The COVID-19 pandemic highlighted various issues regarding healthcare providers’ well-being and their need for employer support.

**Objectives:**

This study aims to show the extent and manner in which end-of-life care providers were supported by their employers during the COVID-19 pandemic, and whether their needs and the support they received matched.

**Design:**

A longitudinal mixed-methods study among healthcare providers delivering end-of-life care during the COVID-19 pandemic.

**Methods:**

Surveys were conducted at four timepoints (*n* = 302), and interviews were conducted at three timepoints (*n* = 17) during the first 18 months of the pandemic. Descriptive analysis was performed on the quantitative data, and thematic analysis was conducted on the interview data.

**Results:**

The survey indicated that healthcare providers’ need for support decreased over the pandemic, yet 18 months after its onset, nearly one in five still needed more support than usual. About one in three healthcare providers felt inadequately supported emotionally across the four periods. Disparities in employer support were found, with fewer healthcare providers receiving support than those who desired it. Interviews revealed that healthcare providers needed their employers to show interest and appreciation and involve them in decision-making. There was also a need for accessible professional support, efforts to reduce the stigma around seeking it, and the facilitation of peer support. In addition, healthcare providers desired practical support during the crisis, such as COVID-19 protection, scheduling, and good information provision.

**Conclusion:**

The COVID-19 pandemic has revealed issues concerning the well-being of healthcare providers. This study gives insight into the amount and type of employer support needed. However, the support provided has often been misaligned with the needs of healthcare providers. As we face another potential healthcare crisis with shortages and high workloads, it is important to maintain focus on the well-being of healthcare providers. Employers must invest in appropriate and effective support measures.

## Plain language summary

(Mis)alignment of employer support and needs of healthcare providers in end-of-life care during a healthcare crisis

The COVID-19 pandemic acted as a pressure cooker, exposing various issues regarding healthcare providers’ wellbeing and their need for employer support. This study aimed to understand how these healthcare providers were supported by their employers and if the support matched their needs.

We conducted surveys with 302 healthcare providers at four different times during the pandemic and interviewed 17 providers at three different times. Our findings showed that the need for support decreased over time, but even 18 months into the pandemic, nearly 20% of providers still needed more support than usual. About one-third of providers felt they were not adequately supported emotionally. Furthermore, not all healthcare providers received the support they wanted from their employer, with fewer providers getting support than those who wanted it.

Our interviews revealed that healthcare providers needed their employers to show interest in them, appreciate their work, and involve them in decision-making. They also wanted easy access to professional support, less stigma around seeking help, and more opportunities for peer support. Practical needs included adequate COVID-19 protection, better scheduling, and clear information.

In conclusion, while the pandemic has emphasized the importance of supporting healthcare providers, the support provided by employers often did not meet their needs. As we face ongoing healthcare challenges, it is important for employers to invest in effective and appropriate support measures to ensure the well-being of healthcare providers.

## Introduction

The healthcare sector is currently facing high levels of work pressure, a problem expected to increase in the future.^1,2^ As a result, the accessibility and quality of care are under pressure.^2^ This is a crisis affecting the entire healthcare system, including palliative care, a sector expected to see increased demand in the coming years.^3,4^ The shortage of healthcare providers (HCPs) is one of the biggest challenges in palliative care,^4^ making it important to retain our palliative care workforce. However, their well-being is currently a concern.

A review of the prevalence of burnout in HCPs providing palliative care reports that most studies found a prevalence of 18% or higher (ranging from 3% to 66%) in 22 different countries.^5^ For the Netherlands specifically, a study shows that 69% of HCPs in palliative care had medium levels of symptoms of burnout and 7% had taken sick leave.^6^ This is especially concerning at a time when these HCPs are critically needed.

The COVID-19 pandemic has revealed both the resilience and vulnerabilities within the healthcare workforce^1^ and acted as a pressure cooker for pre-existing problems, exposing various issues regarding HCPs’ well-being and their need for support.^7^ HCPs who were specifically involved in end-of-life care encountered circumstances that disrupted the normal process of end-of-life care, such as wearing personal protective equipment and limiting contact with patients and relatives, to prevent the spread of the COVID-19 virus.^8–12^ This impacted the well-being of end-of-life care providers.^13^ Studies show that they experienced symptoms such as stress, depression, anxiety, post-traumatic stress, and emotional exhaustion.^13–18^ Studying the well-being and support needs of HCPs during this time of crisis allows us to learn a lot about how to best support HCPs, also in times of a possible next healthcare crisis.

In the literature, four types of support are distinguished: emotional support (e.g., caring and empathy), instrumental support (provision of practical support), informational support (advice and information), and appraisal support (e.g., encouragement)^19^ and it is possible for employers to provide support in these different domains. In Dutch palliative care, a substantial part of HCPs in palliative care needs support at a team and organizational level, with unmet needs from supervisors (25%) and organizations (21%).^20^ This need for support is echoed in various studies that indicate different forms of support from organizations and employers are important during the COVID-19 pandemic.^21–26^ Frontline HCPs’ experiences during the COVID-19 pandemic and previous pandemics confirm this need for support: while HCPs appreciated organizational support, many felt it was insufficient. They anticipated receiving support in exchange for their dedication and sacrifices, but that need was not always fulfilled.^21^ Furthermore, the organization’s understanding of the emotional support needs of staff during COVID-19 positively impacted the resilience of healthcare workers.^22^ Additionally, support and recognition from the healthcare team, government, and community were identified as protective for HCPs’ mental health.^23^ Furthermore, the need for a safe, employee-oriented work environment, good and transparent communication, listening to staff input, and appreciating healthcare workers is important in reducing the mental health burden on healthcare workers during the COVID-19 pandemic. However, the studies do not provide specific strategies for demonstrating appreciation.^24^

Literature shows that employers play a crucial role in providing emotional support to healthcare professionals during the pandemic.^21,23,24^ Despite this, there is a gap in the literature regarding the extent to which HCPs in end-of-life care felt they received the necessary support from their employers. Most studies have focused on the initial stages of the pandemic using cross-sectional designs. However, distress among HCPs persisted beyond the initial months of the pandemic.^13,18^ Prolonged emotional and interpersonal stress on the job can lead to burnout^27^ and subsequent turnover of healthcare workers. In addition, the long-lasting nature of the pandemic and its evolving challenges highlight the need for longitudinal studies. Such studies can capture changes over time and address the shifting needs and support required as we approach the later stages of the pandemic. This study addresses this gap by employing a longitudinal mixed-methods approach to examine the perspectives of HCPs in end-of-life care regarding the support they received from their employers throughout the pandemic. The aim of this study is to show the extent and manner in which end-of-life care providers felt supported by their employers during the COVID-19 pandemic. It examines to what extent their needs and the support offered matched. This information is crucial for better supporting care providers in future crises, such as the impending shortage crisis in healthcare.

## Methods

This study is part of the CO-LIVE study: a mixed-methods study into the experiences of bereaved relatives and HCPs who provided end-of-life care during the COVID-19 pandemic.^9^ In this study, surveys and interviews were utilized at multiple time points during the pandemic among HCPs working in end-of-life care. This approach allowed findings from earlier interviews and surveys from the CO-LIVE study to inspire the questions in subsequent surveys and interviews, giving the research a mixed-methods character. The reporting of this study conforms to the COREQ and STROBE statements^28,29^ (Supplemental Files 1 and 2).

### Population and data collection

#### Surveys

Data was collected from a convenience sample of HCPs that provided end-of-life care (for both COVID-19 and non-COVID-19 patients) during the initial 18 months of the COVID-19 pandemic (March 2020–September 2021). These HCPs came from various professions and from different settings in the Netherlands.

**Figure 1.**
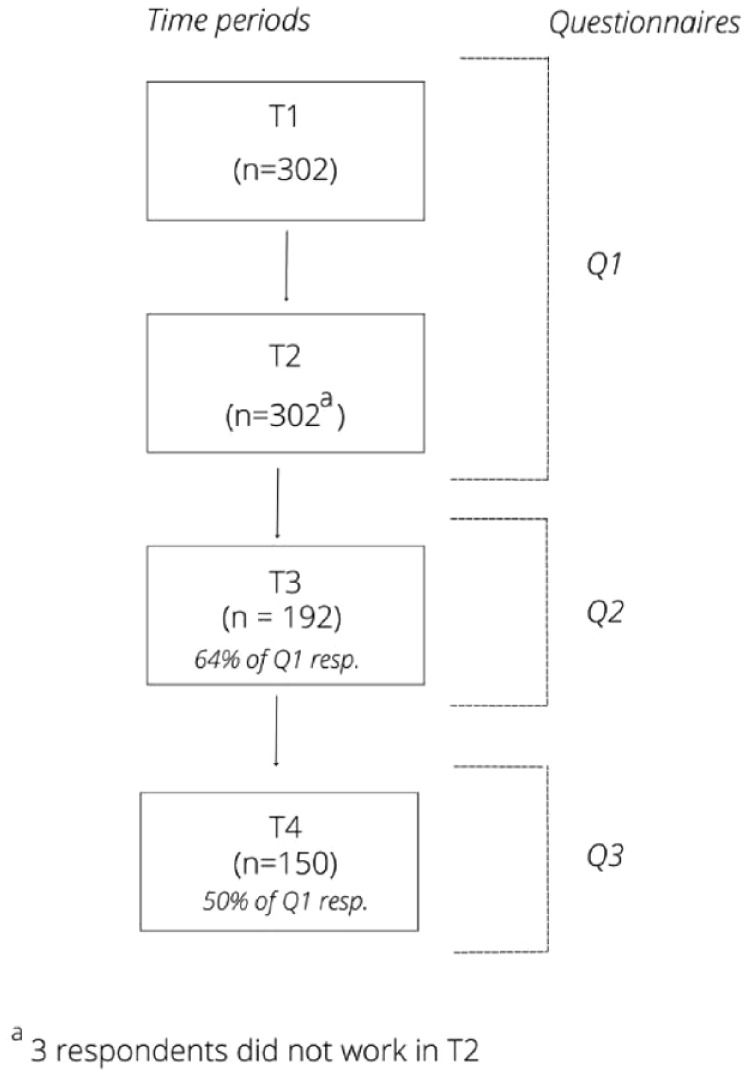
Respondents per time period and questionnaire.

**Figure 2.**
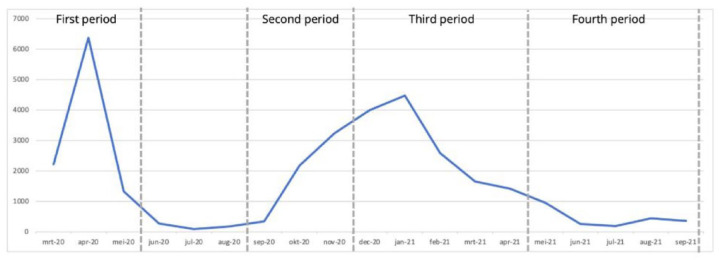
COVID-19 deaths in the Netherlands, march 2020–September 2021.

Data collection covered four time periods, with three questionnaires, Q1, Q2, and Q3 (Figure 1). Q1 was distributed in November 2020 and contained questions about two time periods: March 2020–May 2020 (T1) and September 2020–November 2020 (T2). These periods are considered to be the first wave (T1) and the start of the second wave (T2) of the COVID-19 pandemic in the Netherlands.^30,31^ Q2 was distributed in April 2021 and concerned the period between December 2020 and April 2021 (T3). Q3 was distributed in September 2021 and concerned the time period between May 2021 and September 2021 (T4). Figure 2 shows the number of people who died of COVID-19 in the Netherlands within the four time periods. This provides context about the severity of the pandemic in these researched time periods.^32^

Invitations for Questionnaire 1 (Q1) were sent to end-of-life care providers who had participated in a previous part of the CO-LIVE study.^9^ Additional respondents for Q1 were recruited via (social) media. When respondents indicated in the Q1 questionnaire that we could approach them for another questionnaire, they received an invitation to Q2 and Q3. No other respondents were recruited for Q2 and Q3. Furthermore, respondents of Q1 who missed Q2 could participate in Q3. All questionnaires were designed and distributed via questionnaire software Survalyzer (Survalyzer AG).

#### Interviews

In the survey, respondents were asked whether they would like to participate in the interview study and interview respondents were recruited using maximum variation sampling to ensure diversity in setting and well-being (specifically their Well-Being Index score in T2) via e-mail. An in-depth qualitative longitudinal interview study was conducted with nurses and nurse aides of patients who died during the COVID-19 pandemic. The decision was made to conduct the interview study among nurses because they represented the most vulnerable group. The respondents all had experience with end-of-life care during the pandemic. Some worked in settings specifically focused on end-of-life care, such as hospices, while others worked in institutions where end-of-life care was one aspect of their responsibilities, alongside care for other patients, such as in hospitals. While many HCPs experienced mental health issues in practice,^33,34^ we did not see this represented in our sample. Therefore, we recruited three additional participants who did not participate in the survey, via social media, networks, and previous study participants. Two nurse aides were also recruited from an earlier part of the CO-LIVE study to address the underrepresentation of this profession.^35^ Three rounds of in-depth interviews were held with the participants. The first round of interviews took place from March to May 2021, the second from August to September 2021, and the third from January to April 2022. In total, 43 interviews were conducted with 17 participants.

### Measurements and analysis

#### Surveys

In the surveys, respondents answered questions regarding the need for emotional support and the emotional support that was given to HCPs (T1–T4) during the COVID-19 pandemic. The questions were self-developed and were based on the situation surrounding the pandemic, existing literature, and insights gathered from interviews with HCPs about end-of-life care during the pandemic^8^ (Supplemental Appendix 1).

The questions assessed whether respondents felt they got enough emotional support (using a five-point Likert scale, categorized into three categories: “agree,” “neutral,” and “disagree”). HCPs also indicated if they needed less, the same, or more emotional support than usual, and from whom they got emotional support. In T3 and T4 we added a question from whom HCPs wanted emotional support (Supplemental Appendix 1).

Characteristics of respondents included gender, age, profession, and setting. Setting was categorized into: home, nursing home, hospice facility, other (including for example a GP practice or institutions for people with intellectual disabilities), and multiple settings. Profession of HCPs was divided into three categories: nurses (including registered nurses, nursing aides, and nurse practitioners), physicians (e.g., general practitioners, pulmonary, and geriatric physicians), and others (e.g., spiritual counselors, paramedics, and volunteers). IBM SPSS Statistics 28 (IBM Corporation) was used for descriptive statistical analysis of the data.

#### Interviews

First author MZ, a female medical anthropologist trained in qualitative research, conducted all semi-structured interviews via (video) calls due to COVID-19 measures. A topic list, including questions from the survey responses and new experiences, was used (Supplemental Appendix 2). The interviews explored how nurses interpreted emotional support, felt supported in various ways, and what their needs were. The interviews, conducted in Dutch, lasted between 21 and 67 min and were audio-recorded.

After conducting the interviews, data were transcribed verbatim and analyzed using the qualitative data analysis software MAXQDA (VERBI Software). We followed the principles of inductive thematic analysis focusing on the lived experiences of the respondents.^36,37^ After familiarizing themselves with the data by reading the transcripts, MZ and RP independently coded 16 transcripts from 5 participants to compare codes and minimize bias. MZ then coded the remaining interviews and discussed the codes with RP, BOP, and EW. MZ grouped the codes into overarching themes, with continuous comparison and discussion among MZ, RP, BOP, and EW. Finally, appropriate quotes were selected by MZ and translated by a professional translator.

### Ethical considerations

Study information was provided with each questionnaire, and respondents gave consent before participation. Interview participants gave verbal informed consent for participation and recording. After transcription, audio recordings were deleted, and all data were anonymized to ensure confidentiality. Personal information and transcripts were stored separately, accessible only to researchers. The Medical Ethics Committee Erasmus MC of Rotterdam assessed that the study did not fall under the Medical Research Involving Human Subjects Act (MEC-2020-0254).

## Results

### Characteristics of HCPs

Data of 302 (T1), 299 (T2), 192 (T3), and 150 (T4) respondents is included. The characteristics of the respondents in the different time periods are described in Table 1. Most respondents were women (87.2%–90.1%) and between 46 and 60 years of age (44.9%–55.8%). Over half of the respondents had a nursing background (61.6%–71.8%) and about one-third worked in a hospital (27.4%–30.3%).

**Table 1.** Characteristics of HCPs and end-of-life care during four different time periods (absolute numbers and percentages).

Characteristics	T1	T2	T3	T4
	Q1^a^ *N* = 302 *N* (%)		Q2 *N* = 192 *N* (%)	Q3 *N* = 150 *N* (%)
Gender				
Men	32 (10.8)		19 (9.9)	19 (12.8)
Women	265(89.2)		173 (90.1)	129 (87.2)
Age				
⩽35 years	61 (20.7)		30 (15.9)	13 (8.8)
36–45 years	58 (19.7)		29 (15.3)	23 (15.6)
46–60 years	132 (44.9)		96 (50.8)	82 (55.8)
>60 years	43 (14.6)		34 (18.0)	29 (19.7)
Profession				
Nurse	216 (71.8)		129 (68.8)	90 (61.6)
Physician	40 (13.2)		24 (12.8)	22 (15.1)
Other	45 (15.0)		35 (18.6)	34 (23.4)
Setting				
Home	47 (15.8)		33 (17.6)	20 (13.7)
Nursing home	64 (21.5)		34 (18.1)	29 (19.9)
Hospital	90 (30.3)		53 (28.2)	40 (27.4)
Hospice facility	54 (18.2)		38 (20.2)	29 (19.9)
Other	17 (5.7)		13 (6.9)	15 (10.3)
Multiple	25 (8.4)		17 (9.0)	13 (8.9)

Number of missings range (over Q1–Q3): gender (0–5), age (3–8), profession (0–4), setting (3–5).

a Q1 was distributed in T2 and contained questions about both T1 and T2.

HCP, healthcare provider.

The characteristics of the 17 interview respondents are included in Supplemental Appendix 2. All interview respondents were women, with 13 being nurses and 4 being nursing aides. Nurses and nurse aides were represented across various settings, including hospitals, ICUs, nursing homes, home care, and hospice facilities, and most participated in three interviews.

### Emotional support needs

Table 2 shows (the need for) emotional support experienced by HCPs per time period. The need for more emotional support than usual was highest in T1 at 46.3%, decreased to 27.3% in T2, 28.7% in T3, and was lowest in T4 at 18.5%. Regarding receiving emotional support, in T1, 64.4% of HCPs felt they received enough emotional support, with similar percentages in T2 (63.3%), T3 (63.1%), and T4 (57.7%).

**Table 2.** (The need for) Emotional support experienced by HCPs during the COVID-19 pandemic.

Variables	T1 *N* = 302 *N* (%)	T2 *N* = 299 *N* (%)	T3 *N* = 192 *N* (%)	T4 *N* = 150 *N* (%)
Needed emotional support				
Less than usual	2 (0.7)	10 (3.4)	9 (4.8)	4 (2.7)
The same as usual	159 (53.0)	206 (69.4)	125 (66.5)	115 (78.8)
More than usual	139 (46.3)	81 (27.3)	54 (28.7)	27 (18.5)
Got enough emotional support				
Agree	194 (64.4)	190 (63.3)	118 (63.1)	83 (57.7)
Neutral	72 (23.9)	81 (27.0)	48 (25.7)	47 (32.6)
Disagree	35 (11.6)	29 (9.7)	21 (11.2)	14 (9.7)
Received emotional support from				
No one	4 (1.3)	5 (1.7)	2 (1.1)	5 (3.4)
Partner/friends/family	247 (81.8)	259 (85.8)	166 (88.3)	128 (87.7)
Colleagues	256 (84.8)	253 (83.8)	159 (84.6)	118 (80.8)
Employer	115 (38.1)	114 (37.7)	50 (26.6)	26 (17.8)
Professional	7 (2.3)	15 (5.0)	18 (9.6)	14 (9.6)
Other	12 (4.0)	15 (5.0)	11 (5.9)	4 (2.7)
Wanted emotional support from				
No one			18 (9.6)	12 (8.2)
Partner/friends/family			141 (75.0)	106 (72.6)
Colleagues			135 (71.8)	103 (70.5)
Employer			75 (39.9)	51 (34.9)
Professional			23 (12.2)	11 (7.5)
Other			7 (3.7)	3 (2.1)

HCP, healthcare provider.

Most HCPs received emotional support from partners, friends, or family and colleagues with percentages higher than 80% across all periods. However, the percentage of HCPs receiving emotional support from their employers was lower: highest at 38.1% in T1 and dropping to 17.8% in T4. The majority of HCPs desired support from their partners, friends, and family (T3: 75.0% and T4: 72.6%) and their colleagues (T3: 71.8% and 70.5%). Over one-third of HCPs indicated that they wanted support from their employer (T3: 39.9% and T4: 34.9%). The category of “other” consisted of example religion, patients, and their own healthcare workers (such as a physiotherapist).

**Figure 3.**
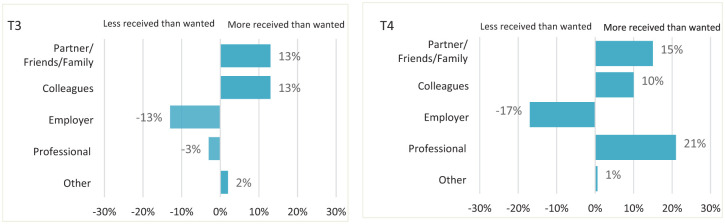
Discrepancies between wanting and receiving emotional support.

Disparities were found between the emotional support HCPs wanted and what they actually received. Figure 3 illustrates these differences, revealing positive differences in emotional support from partner/friends/family and colleagues during T3 and T4, suggesting more support received than desired and there was a negative difference of −13.3% in support from employers, indicating fewer HCPs received the support they sought. This pattern persisted in T4, where the difference was −17.1%.

In the interviews, nurses also shared their satisfaction with the support from their employer. Most nurses expressed a need for support from their employers during the COVID-19 pandemic. While some were very satisfied with the support provided, others felt it was inadequate and mentioned that they missed that support.

And then the manager says, “But you can cope with this.” But that’s not what it’s about. Sure we can cope, but we still need support sometimes, right. (R6: nurse, nursing home)

Many nurses indicated that they felt supported by their employers in the initial period of the pandemic, but that the support decreased as time progressed:

But I don’t think there was so much compared with the first wave. That was like a coccoon: all the operations were cancelled, we had all the facilities, there were suddenly plenty of opportunities to share things with each other. And that was less the case after the summer. (R8: nurse, hospital)

#### Emotional support needs per setting

Table 3 shows that in T1, HCPs in nursing homes most often indicated that they needed more support than usual (60.9%). This was followed by hospitals, where 54.4% of HCPs felt the need for more support than usual. In T2, while the overall percentage decreased, similar patterns were observed across different settings, with HCPs in nursing homes, hospitals, and home care reporting the highest need for support. By T3 and T4, the need for extra support decreased across all settings.

**Table 3.** (The need for) Emotional support from employers experienced by HCPs during the COVID-19 pandemic per work setting.

Variables	T1 *N* = 302 *N* (%)	T2 *N* = 299 *N* (%)	T3 *N* = 192 *N* (%)	T4 *N* = 150 *N* (%)
Need emotional support more than usual				
Total	139 (46.3)	81 (26.4)	54 (28.7)	27 (18.5)
Home care	17 (36.2)	13 (28.3)	10 (30.3)	4 (20.0)
Nursing home	39 (60.9)	18 (37.5)	12 (35.3)	4 (13.8)
Hospital	49 (54.4)	23 (26.1)	19 (35.8)	10 (25.0)
Hospice	17 (31.5)	10 (20.0)	4 (10.5)	4 (13.8)
Other	5 (29.4)	6 (16.7)	4 (30.8)	3 (20.0)
More than one setting	11 (44.0)	10 (38.5)	5 (29.4)	2 (15.4)
Got enough emotional support				
Total	194 (64.4)	190 (63.3)	118 (63.1)	83 (57.7)
Home care	32 (68.1)	30 (65.2)	22 (66.7)	12 (60.0)
Nursing home	32 (50.0)	25 (51.0)	18 (52.9)	11 (39.3)
Hospital	63 (70.0)	55 (62.5)	25 (48.1)	18 (46.2)
Hospice	37 (69.8)	33 (64.7)	33 (86.8)	22 (75.9)
Other	11 (64.7)	27 (73.0)	8 (61.5)	9 (60.0)
More than one setting	16 (64.0)	19 (73.1)	12 (70.6)	11 (84.6)
Got emotional support from employer				
Total	155 (38.1)	114 (37.7)	50 (26.6)	26 (17.8)
Home care	21 (44.7)	21 (45.7)	10 (30.3)	8 (40.0)
Nursing home	22 (34.4)	17 (34.0)	5 (14.7)	4 (13.8)
Hospital	33 (36.7)	24 (27.3)	12 (22.6)	4 (10.0)
Hospice	24 (44.4)	23 (45.1)	21 (55.3)	7 (24.1)
Other	5 (29.4)	18 (47.4)	0 (0.0)	1 (6.7)
More than one setting	9 (36.0)	10 (38.5)	2 (11.8)	2 (15.4)
Wanted emotional support from employer				
Total			75 (39.9)	51 (34.9)
Home care			9 (27.3)	8 (40.0)
Nursing home			18 (52.9)	10 (65.5)
Hospital			18 (34.0)	12 (30.0)
Hospice			21 (55.3)	11 (37.9)
Other			3 (23.1)	5 (33.3)
More than two settings			6 (35.3)	5 (38.5)

The emotional support HCPs got from their employer varied by worksetting. The percentage of HCPs that received support from their employer was highest in all four time periods in hospices (ranging from 24.1% to 45.1%) and in home care (ranging from 30.3% to 45.7%) and lower in nursing homes (ranging from 13.8% to 34.4%) and in hospitals (ranging from 10.0% to 36.7%).

In both T3 and T4, the need for emotional support from employers was highest in nursing homes (T3: 52.9% and T4: 65.5%) and hospices (T3: 55.3% and T4: 37.9%). HCPs in home care and hospital showed less desire for emotional support from their employer with 27.3% and 34.0% in T3 and 40.0% and 30.0% respectively in T4.

**Figure 4.**
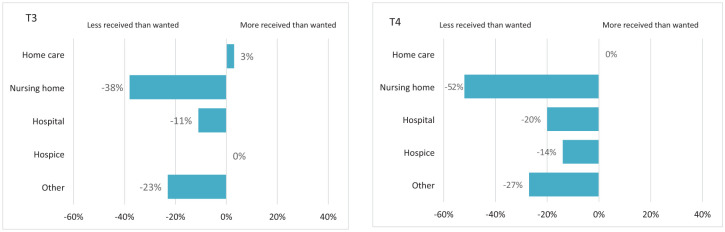
Discrepancies between wanting and receiving emotional support from employers per setting.

Figure 4 illustrates that during T3, fewer HCPs received the support they desired in nursing homes (−38.2%) and hospitals (−11.4%). These discrepancies persisted into T4, with deficits in support observed in nursing homes (−51.7%) and hospitals (−20.0%).

### How nurses want to be supported by their employers

It is clear that HCPs seek support from their employers. The interviews were conducted to understand what nurses considered emotional support and investigated how nurses prefer to receive support. The findings showed that emotional support was interpreted broadly, encompassing various forms of support. Three themes were identified about the needs for support: seeing HCPs, facilitating emotional support, and facilitating practical support (Table 4).

**Table 4.** Themes.

Themes	Subthemes	Codes
Seeing HCPs	Taking interest in experiences	Active listening when HCPs share experiences and feelings
		Invite HCPs to share experiences and feelings
		Take feelings, experiences, and problems seriously
	Appreciating HCPs	Actually articulate appreciation
		Provide them with a small gesture
		Acknowledge and recognize the seriousness of challenges and concerns of the crisis
		Optimize employment conditions
		Protect them from overworking
	Including HCPs in decision-making	Include HCPs in decisions concerning their units/departments
		Include HCPs in decisions concerning local COVID-19 measures
		Invite them to evaluate after
Facilitating emotional support	Professional support	Make professional accessible
		Address the taboo surrounding seeking professional help
		Exert more pressure to encourage the use of these services
	Peer support	Create opportunities to share experiences among each other
		Provide tools for those who provide peer support
		Facilitate work-related meetings to discuss challenging situations with colleagues
		Facilitate informal interactions
Facilitating practical support	Protecting HCPs	Protect HCPs against COVID-19 infections
	Information provision	Provide clear information about the circumstances
	Support in difficult situations	Allow them to rely on supervisors in difficult situations
	Creating a good work environment	Flexible scheduling
		Provide an equitable distribution of the type of work and the opportunity of doing other tasks
		Organize facilities to make work environment more pleasant

HCP, healthcare provider.

### Seeing nurses

Nurses often felt unseen by their employers, despite it being important for them to feel supported. They emphasized that being seen required employers to show interest in their experiences, show appreciation, and involve them in decision-making.

#### Taking interest in experiences of nurses

Nurses needed their employers to not only listen actively when they talked about their experiences and feelings but also invite them to share, rather than having to initiate these conversations themselves. This need was evident whether interactions were individual or group-based, formal or informal, and both during the peaks of the pandemic and after those peaks. Additionally, some nurses felt they lacked emotional support when they did share, as they felt their problems were not taken seriously.

Look, it [getting a burnout] could have been prevented—I’m sure of that. Because I did tell them that I wasn’t doing so well, but they kind of dismissed it. (R13: nurse aid, nursing home)

#### Appreciating nurses

Feeling appreciated was very important for nurses, yet they often didn’t experience this. When they did feel genuinely appreciated, they always found this experience to be very positive.

For a while, we had this team leader who showed appreciation for everything and everyone, so that was really nice. That gave us an incredible boost because everything you do is actually appreciated. Which is fantastic. (R6: nurse, nursing home)

Nurses needed employers to articulate their appreciation. They wanted to hear (or read) that their work was recognized and valued. Furthermore, although nurses did not find it necessary, they could value a small gesture (such as a small present or postcard) from time to time. Nurses also stressed the importance of employers acknowledging and recognizing the seriousness of their challenges and concerns. They expressed feeling unappreciated when employers focused only on the positive aspects of their work.

It boils down to appreciation, appreciation that you’re there, plus room for. . . [. . .] for your boss to express that because—you know—it’s tough work you’re doing; it’s difficult. (R6: nurse, nursing home)

Some interviewed nurses, particularly nurses in the ICU, were dissatisfied with their employment conditions, such as their salary, which contributed to their feelings of not being appreciated by their employers. In addition, nurses felt appreciated when their supervisor protected them from overwork, with some noting that this support helped prevent burnout symptoms.

And also having your team manager give you the space [. . .] to look after yourself properly. Because the healthcare culture is one of [. . .] doing a lot of stuff at home too, reading through files before coming into work. She said you shouldn’t be doing that, as it’s part of your job. So you don’t need to already be doing that stuff at home; you can just do all that when you get to work. And perhaps the clients need to wait a bit longer, but so be it. That’s also really nice as I think it shows appreciation. (R6: nurse, nursing home)

#### Including nurses in decision-making

Some nurses felt overlooked when decisions concerning their units or COVID-19 measures, such as local regulations on personal protective equipment usage or significant decisions such as departmental reassignments or hiring, were made without their knowledge or consultation. For instance, a nurse told how she had to learn from television that her department might be repurposed if the COVID-19 situation worsened.

And that led to a lot of anger and disappointment, as well as a sense of panic—how are we going to care properly for all those people? I did feel the blood drain from my face for a moment then, like “What’s going on: this just got decided?” We do get that feeling sometimes, that these decisions are just taken without considering our views at all. Of course I realize you can’t go and ask all sixty nurses for their opinions, but there was no communication about this at all. And we ended up learning what was going to happen from the TV. (R14: nurse, hospital)

Furthermore, some nurses missed the opportunity to discuss with their employers about what happened, what decisions were made, and their perspectives on how improvements could be made.

### Facilitating emotional support

Nurses emphasized the need for their employers to facilitate emotional support. This could be achieved through arranging professional assistance or facilitating peer support.

#### Professional support

All interviewed nurses noted that during the pandemic, they had access to professional support such as helplines, psychologists, social workers, or spiritual counselors through their organization. They appreciated having these options available, although one nurse mentioned that it started too late. However, most nurses did not utilize these services, either because they preferred to receive support in other ways or because they felt the care was not easily accessible:

Because I never did see them [the psychologists]. How was I supposed to know they were in some separate office. . . because nobody ever went there. So you just had to know where it was they were. Well, one of them handled things a bit better and turned up occasionally in the coffee corner and gave us some general tips—things you could use, let’s say. (R2: nurse, hospital)

Nurses also felt that there was a taboo surrounding seeking professional help, partly due to the persevering nature of HCPs. A nurse expressed a desire for the organization to address this issue.

Because we’re all so tough, pretending it won’t ever happen to me. So there is this huge taboo—at any rate I feel there’s still a huge taboo—on seeing a psychologist, like “I don’t need this and what use will it be to me?” I think we should do much more to tackle that as an organization, and as a team too. (R15: nurse ICU)

Although most interviewed nurses said they appreciated the voluntary nature of professional help, a few nurses felt that the management should have exerted more pressure to encourage the use of these services.

#### Peer support

While nurses appreciated professional support, they often felt a stronger need for peer support, valuing conversations with others who understood their situation. However, COVID measures reduced opportunities for contact among colleagues.

Nurses therefore needed their employers to create opportunities for colleagues to share their experiences with each other, whether online or in person. Nurses described managers conducting daily check-ins with the scheduled team and facilitating sharing with each other. Some nurses expressed they wanted to provide peer support but felt they lacked the necessary tools, although some mentioned available training for this purpose.

Nurses emphasized the importance of not only sharing feelings and experiences but also having work-related meetings to discuss challenging situations with colleagues. Some nurses really missed the opportunity for those formal conversations in the hospice where she worked:

What I didn’t like at all [. . .] was that the peer review sessions stopped. I thought at the time, and I said it, that I thought it was stupid; surely they could find a way to continue with the peer review sessions, especially now. I mean in a hospice in particular, with lots of cases and all those restrictions. But they didn’t do that. And I thought then: don’t get so submerged in being overworked and keeping everything going that you forget yourself. I mean, I pointed out to them: don’t forget that. (R11: nurse, home care & hospice facility)

Nurses also indicated that, in addition to the more formal moments together, they also desired informal interactions, although this was often completely disrupted. They mentioned that especially during this time, it was important to connect with each other in other ways, such as online or in small groups, and to continue planning these moments, even though they were unsure if they would actually happen.

### Facilitating practical support

The challenging situation called for practical support for nurses, and at times nurses felt they did not receive enough practical support to cope with the situation.

#### Protecting nurses

At times, nurses felt unsupported because they believed their employer did not adequately protect them against COVID-19 infections, such as through vaccinations, personal protective equipment, or enforcement of safety protocols:

More and more groups were being given priority [for the vaccinations]. I did find that a tricky issue. Partly because I did get COVID. I think I caught it in the hospital; I don’t know for certain, but that’s the most likely. So then I think. . . well, sometimes it feels a bit as if we were left in the lurch. (R8: nurse, hospital)

#### Information provision

Nurses appreciated being well-informed about the circumstances, typically through (online) updates.

#### Support in difficult situations

Nurses indicated that they wanted to be able to rely on their supervisors in difficult situations (such as discussions with family members), even if the supervisors were not physically present. For example, a nurse aid dealing with a family member who disagreed with the COVID-19 measures:

So there came a point where we simply explained the situation to our boss, saying this is what’s going on, this is what we’ve done so far, can you please take over because I’m not getting anywhere. And my old boss was at home at the time, spending as little time as possible at the workplace, so they phoned the son and it was sorted immediately. There was no problem at all after that. (R5: nurse aid, nursing home)

#### Creating a good work environment

In these circumstances, it was important to create the best possible working environment for nurses. This included aspects such as scheduling, where flexibility with hours could be accommodated and extra shifts could be arranged as requested by nurses.

And then they said, “No, we can’t take on an extra person—we’re not allowed to have extra staff because we don’t have the money for that.” And then you do miss that support, you feel that lack of support. Fortunately it was quiet that Saturday. But the Sunday was a disaster [. . .]. Then we got an extra person from the Monday. But then they don’t listen to you again, so you’re constantly playing catch-up. (R5: nurse aid, nursing home)

Nurses also said that the same people were consistently assigned to COVID-19 care and expressed their need for a more equitable distribution of the type of work they do and the opportunity to engage in other tasks (mainly in hospitals and nursing homes). But nurses also discussed organizing facilities to make the work environment more pleasant, such as providing meals during the peak of the COVID-19 pandemic.

## Discussion

This longitudinal mixed-method study shows the extent to which end-of-life care providers needed support, the extent they felt supported by their employers in particular during the pandemic, and what their needs regarding employer support were. The survey indicated that, over the course of the pandemic, HCPs reported a decreasing need for additional support, yet nearly one in five still required more support than usual 18 months after the start of the pandemic. About one in three HCPs did not feel adequately supported emotionally overall during the four time periods. HCPs reported receiving the most support from their partners, family, friends, and colleagues (all above 80% in all time periods). In fact, the number of providers who received support from partners, family, friends, and colleagues exceeded those who wanted it. However, there were disparities between the emotional support HCPs desired from their employers and what they actually received, especially noticeable in nursing homes. Qualitative findings highlighted that many interviewed nurses indicated a need for increased support from their employers during the COVID-19 pandemic, although they often did not receive it. In addition, the qualitative results shed light on the nature of the support desired. The results revealed that emotional support was understood in a broad sense, including several different types of support.

The study emphasizes the importance of employers showing interest in, appreciating, and involving nurses in decision-making processes. There was also a need for accessible professional support and efforts to reduce the taboo around seeking it. An even greater need was for peer support, with nurses wanting employers to facilitate both formal and informal opportunities to receive and give this form of support. In addition, nurses desired practical support during the crisis, such as protection against COVID-19 infections, scheduling, and good information provision.

### Persistent need for support during and after the pandemic

Due to the longitudinal nature of this study, we can see in the quantitative data that there was still a need for support 18 months after the start of the pandemic but this support was often not received. At a time when appreciation, recognition, and attention from people outside the healthcare sector decreased (“pandemic fatigue”^38^) after the first wave, it is important that employers maintain focus on support. Although some HCPs in the interviews indicated that they needed less support toward the end of the pandemic, others missed the support they received at the beginning. There was also a need for aftercare following the chaos from the beginning of the pandemic. It is important to continue researching the support needs of HCPs, even as more time passes since the pandemic. Literature shows that healthcare workers still experience reduced well-being long after the pandemic began.^39–42^

### Broad interpretation of emotional support

It is interesting that the interview respondents had a broad interpretation of emotional support and felt emotionally supported through other forms of help, such as practical support. Ultimately, practical support also contributed to their mental well-being. For example, they felt less fearful when adequately protected against COVID-19 infections, highlighting the overlap between different types of support. The identified themes, namely seeing HCPs, facilitating professional support, and practical support, align closely with findings from other studies on support during the COVID-19 pandemic.^21–26^

### Similar support needs across HCPs

While our focus was on end-of-life care, the support needs described in the literature seem to be broadly similar across healthcare contexts.^21–25^ This may partly be due to the context in which these HCPs work. Some were employed in settings focused exclusively on end-of-life care, such as hospice facilities, while others worked in environments where end-of-life care was just one part of their broader responsibilities. While end-of-life care during the pandemic was particularly intense and providers faced specific challenges, which are not discussed in this article, the overall needs for support were general and could apply to those specific challenges. The distinction lies in the types of work-related issues for which HCPs seek help, rather than in how they prefer to receive support.

### Appreciation from employers

A significant theme in this study is appreciation. While this theme is also frequently mentioned in the literature,^21–25^ there is little guidance on how to effectively show appreciation.^24^ In the interviews, concrete examples were provided on what this appreciation looks like (see also Table 4). What we noticed is that the concept of appreciation was interpreted very broadly by the interviewed nurses, with many different ways in which nurses felt valued. This indicates a wide, subjective experience. Appreciation is not just about verbal recognition; nurses also feel appreciated when they are helped, given space, and protected by their employer. In the literature, four different types of support are distinguished: emotional, instrumental, informational, and appraisal.^19^ However, there seems to be an overlap between appraisal support and the other forms of support. If working conditions are poor (instrumental) or if people do not feel taken seriously (emotional), it affects their sense of appreciation. The notion that appreciation can vary for everyone and that there are many different forms of it is also found in a study among oncology clinicians, though not necessarily in the context of the pandemic.^43^ What is often missing from other studies is the simple act of acknowledging that the situation is challenging and difficult. In the current study, nurses mention it is not always appropriate to approach everything with a positive outlook. To truly appreciate HCPs, it is essential to listen to them and understand what is happening on the work floor, including visiting the departments to witness firsthand the difficult and heavy situations they face.

### Accessible professional support

Furthermore, the literature extensively discusses the implementation of visible, accessible professional help, such as social workers and psychologists, and this support was indeed available in many places.^21,23,24^ However, it would be interesting to examine the extent to which this care was actually utilized during the crisis. Despite its accessibility, the taboo surrounding seeking help still plays an important role among HCPs due to the perceived or internalized stigma.^44,45^ If there’s a perceived taboo, professional help isn’t truly accessible. It is important to explore how a culture can be created where HCPs feel comfortable and willing to utilize the offered professional support. On the other hand, since people already find it difficult to seek help, making support services more accessible could prove beneficial. Instead of being secluded in a private room, offering support in the form of a cup of tea at a communal table during peak times could foster informal chats. This approach, as indicated by respondents, might encourage more HCPs to take advantage of the support offered, reducing the barrier posed by the taboo associated with seeking help.

### Peer support

Additionally, in our own study, nurses preferred peer support over professional support, which is also confirmed by other studies.^46^ It would be valuable to further investigate what specific forms of peer support HCPs need during a crisis such as the COVID-19 pandemic. Literature also distinguishes peer support between emotional support, informational support, and appraisal.^47^ In our study, it seems particularly important for nurses to connect with peers who are in the same situation, as they feel misunderstood by others. Consequently, there is a pronounced need for emotional and appraisal support among HCPs. However, nurses also needed informational support in the form of work-related meetings such as intervision and moral case deliberation to cope with the challenges of caregiving during the pandemic. It was important for nurses that these meetings continued, even at a time when such gatherings were often avoided due to social distancing. However, this preference for peer support can sometimes feel contradictory. This is because it involves receiving help from colleagues who are also struggling, may not know how to provide support, or are already experiencing high work pressure and have limited time. We need to find a method to ensure that employees can offer peer support without suffering themselves. Beyond employers facilitating gatherings, it might be beneficial to train employees in peer support, emphasizing their own well-being while providing that support.

### Strengths and limitations

A strength of this study is its comprehensive overview of end-of-life care during the pandemic, as it encompasses multiple healthcare settings and professions. The longitudinal design, with data collected up to 18 months after the pandemic’s onset, further enhances its thoroughness. Due to COVID-19 measures, all interviews were conducted via (video) call. This could have affected the depth of the interviews by hampering building rapport with respondents. Despite this, we found respondents eager to share their experiences, and we believe the interviews maintained depth comparable to our usual face-to-face interactions. In the later stages of the study, no new topics emerged compared to earlier interviews. However, the complexity and rapid evolution of the COVID-19 situation across different healthcare settings mean we cannot be certain we reached saturation. This study may have underrepresented respondents in severe emotional distress, as they might have been less likely to complete the (follow-up) questionnaires. Consequently, those who were doing relatively well might be overrepresented, potentially leading to an underestimation of distress and therefore the need for support among HCPs. In addition, there could have been recall bias in the initial questionnaire, which asked about an earlier time period. However, given the exceptional nature of the pandemic’s onset, respondents might recall their feelings during that time quite accurately. Another limitation of this study is that we only asked participants whom they wanted help from in T3 and T4. It would be interesting to see if there were also discrepancies between the desired and received support in T1 and T2. Furthermore, we only included nurses in our interview study. We chose this approach to narrow our focus and because the initial survey indicated that nurses’ well-being was most at risk. It would be interesting to investigate whether these interview results also apply to other HCPs.

## Conclusion

The COVID-19 pandemic has acted as a pressure cooker, revealing substantial issues concerning the well-being of healthcare workers and their need for employer support. This study demonstrates an unfulfilled need for support for healthcare workers; even 18 months after the pandemic began, one in five healthcare workers still required more support than usual. However, the support provided by employers has often been insufficient and misaligned with the needs of these workers. This study has formulated specific recommendations based on the needs of healthcare workers. As we face another potential healthcare crisis with shortages and high workloads, it is important to maintain focus on the well-being of our healthcare workers. Employers must invest in appropriate and effective support measures.

## Supplemental Material

sj-docx-1-pcr-10.1177_26323524241308268 – Supplemental material for (Mis)alignment of employer support and needs of healthcare providers in end-of-life care during a healthcare crisis: a longitudinal mixed-method study during the COVID-19 pandemic (the CO-LIVE study) Supplemental material, sj-docx-1-pcr-10.1177_26323524241308268 for (Mis)alignment of employer support and needs of healthcare providers in end-of-life care during a healthcare crisis: a longitudinal mixed-method study during the COVID-19 pandemic (the CO-LIVE study) by Masha S. Zee, H. Roeline Pasman, Erica Witkamp, Yvonne N. Becqué, Anne Goossensen and Bregje D. Onwuteaka-Philipsen in Palliative Care and Social Practice

sj-docx-2-pcr-10.1177_26323524241308268 – Supplemental material for (Mis)alignment of employer support and needs of healthcare providers in end-of-life care during a healthcare crisis: a longitudinal mixed-method study during the COVID-19 pandemic (the CO-LIVE study) Supplemental material, sj-docx-2-pcr-10.1177_26323524241308268 for (Mis)alignment of employer support and needs of healthcare providers in end-of-life care during a healthcare crisis: a longitudinal mixed-method study during the COVID-19 pandemic (the CO-LIVE study) by Masha S. Zee, H. Roeline Pasman, Erica Witkamp, Yvonne N. Becqué, Anne Goossensen and Bregje D. Onwuteaka-Philipsen in Palliative Care and Social Practice

sj-docx-3-pcr-10.1177_26323524241308268 – Supplemental material for (Mis)alignment of employer support and needs of healthcare providers in end-of-life care during a healthcare crisis: a longitudinal mixed-method study during the COVID-19 pandemic (the CO-LIVE study) Supplemental material, sj-docx-3-pcr-10.1177_26323524241308268 for (Mis)alignment of employer support and needs of healthcare providers in end-of-life care during a healthcare crisis: a longitudinal mixed-method study during the COVID-19 pandemic (the CO-LIVE study) by Masha S. Zee, H. Roeline Pasman, Erica Witkamp, Yvonne N. Becqué, Anne Goossensen and Bregje D. Onwuteaka-Philipsen in Palliative Care and Social Practice

sj-docx-4-pcr-10.1177_26323524241308268 – Supplemental material for (Mis)alignment of employer support and needs of healthcare providers in end-of-life care during a healthcare crisis: a longitudinal mixed-method study during the COVID-19 pandemic (the CO-LIVE study) Supplemental material, sj-docx-4-pcr-10.1177_26323524241308268 for (Mis)alignment of employer support and needs of healthcare providers in end-of-life care during a healthcare crisis: a longitudinal mixed-method study during the COVID-19 pandemic (the CO-LIVE study) by Masha S. Zee, H. Roeline Pasman, Erica Witkamp, Yvonne N. Becqué, Anne Goossensen and Bregje D. Onwuteaka-Philipsen in Palliative Care and Social Practice

sj-docx-5-pcr-10.1177_26323524241308268 – Supplemental material for (Mis)alignment of employer support and needs of healthcare providers in end-of-life care during a healthcare crisis: a longitudinal mixed-method study during the COVID-19 pandemic (the CO-LIVE study) Supplemental material, sj-docx-5-pcr-10.1177_26323524241308268 for (Mis)alignment of employer support and needs of healthcare providers in end-of-life care during a healthcare crisis: a longitudinal mixed-method study during the COVID-19 pandemic (the CO-LIVE study) by Masha S. Zee, H. Roeline Pasman, Erica Witkamp, Yvonne N. Becqué, Anne Goossensen and Bregje D. Onwuteaka-Philipsen in Palliative Care and Social Practice

sj-pdf-6-pcr-10.1177_26323524241308268 – Supplemental material for (Mis)alignment of employer support and needs of healthcare providers in end-of-life care during a healthcare crisis: a longitudinal mixed-method study during the COVID-19 pandemic (the CO-LIVE study) Supplemental material, sj-pdf-6-pcr-10.1177_26323524241308268 for (Mis)alignment of employer support and needs of healthcare providers in end-of-life care during a healthcare crisis: a longitudinal mixed-method study during the COVID-19 pandemic (the CO-LIVE study) by Masha S. Zee, H. Roeline Pasman, Erica Witkamp, Yvonne N. Becqué, Anne Goossensen and Bregje D. Onwuteaka-Philipsen in Palliative Care and Social Practice

## Declarations

**Ethics approval and consent to participate:** Study information was provided with each questionnaire, and respondents gave consent before participation. Interview participants gave verbal informed consent for participation and recording. The Medical Ethics Committee Erasmus MC of Rotterdam assessed that the study did not fall under the Medical Research Involving Human Subjects Act (MEC-2020-0254).

**Consent for publication:** Not applicable.

**Availability of data and materials:** The datasets used and/or analyzed during the current study are available from the corresponding author on reasonable request.
