# Supplementary material for: (Mis)alignment of employer support and needs of healthcare providers in end-of-life care during a healthcare crisis: a longitudinal mixed-method study during the COVID-19 pandemic (the CO-LIVE study)
Source: Palliat Care Soc Pract. 2025 Oct 8;19:26323524241308268. doi: 10.1177/26323524241308268 (PMC12511713; doi:10.1177/26323524241308268)
Supplement: sj-docx-1-pcr-10.1177_26323524241308268 – Supplemental material for (Mis)alignment of employer support and needs of healthcare providers in end-of-life care during a healthcare crisis: a longitudinal mixed-method study during the COVID-19 pandemic (the CO-LIVE study) [file sj-docx-1-pcr-10.1177_26323524241308268.docx]

| **Appendix 1: Original (translated) and recoded questions in the questionnaire** | | |
| --- | --- | --- |
| **Variable** | **Original question** | **Recoded** |
| **Gender** | **What is your gender?**   - Male - Female - Other - I would rather not say |  |
| **Age** | **What is your age?** | **Categorized**  *⩽35 years*  *36-45 years*  *46-60 years*  *>60 years* |
| **Profession** | **In what role were you caring for patients?**  Nursing assistant  Nurse  Physician assistant  General practitioner  Elderly care physician  Other medical specialty, namely:  ___________________________________  Other care professional, namely:  __________________________________  Volunteer | **Categorized**   - Nurse   *Nurse aides, nurse, physician assistant*   - Physician  *General practitioner, elderly care physician, physician with a different specialism* - Other  *Other healthcare professional, volunteer* |
| **Setting** | **In which setting did you provide care?**  *(more than one answer possible)*  At home / community  In a hospital  At an ICU  At a ward for Corona patients (no ICU)  At another ward  In a care home / nursing home  At a ward for Corona patients  At another ward  In a hospice facility (not specifically for Corona patients)  Other (please specify): _______________________________ | **Categorized**   - Home   *At home/community*   - Hospital   *In a hospital (ICU/at ward for COVID patients, at another ward)*   - In a care home / nursing home   *(At a ward for COVID patients, at another ward)*   - Hospice facility   *For COVID patients, not for COVID patients*   - Other   *Other*   - More than one |
| **Needed emotional support** | How much emotional support did you need during [*earlier defined period]*?  ☐ More than usual  As much as usual  ☐ Less than usual |  |
| **Got enough emotional support** | Please rate to what extent you agree with the follow statements when you think about how you felt during [*earlier defined period]*?  I received sufficient emotional support.  Strongly disagree  Disagree  Neutral  Agree   Strongly agree  Don’t know | **Categorized**   - Disagree   *Strongly disagree, disagree*   - Neutral *Neutral* - Agree   *Agree, strongly agree* |
| **Received emotional support from** | From whom did you receive emotional support during [*earlier defined period]*? (more than one answer possible)  ☐ I did not receive emotional support  ☐ From my partner/friends/family  ☐ From my colleagues  ☐ From my employer  ☐ From a professional  ☐ Other, namely (please specify) ____________________________ |  |
| **Wanted emotional support from** | From whom did you want to receive emotional support during [*earlier defined period]*? (more than one answer possible)  ☐ From no one  ☐ From my partner/friends/family  ☐ From my colleagues  ☐ From my employer  ☐ From a professional  ☐ Other, namely (please specify) ____________________________ |  |

**Appendix 2. Interview Topic List**

**Can you tell us about how your work has been since the outbreak of COVID-19?**

- How have you experienced this period?
- Specifically regarding end-of-life care
- Comparison with the first wave (T1) or the last time we spoke

**Can you give an example of a situation where you found end-of-life care challenging/touching/memorable during this period?**

- What made it that way?
- Did the situation present a dilemma?
  - Did this situation cause doubt about what the right thing to do was?
- How did you handle this situation?
- How do you handle dilemmas in your work?
- What helps you in these situations?

**Based on the 'complaints' in the survey (emotionally challenging, need for support)**

- Check if the situation from the survey is still accurate (e.g., in the survey you mentioned xx, yy, zz. Can you elaborate?)
  - Can you give an example?
  - Cause/causes?
  - To what extent has (the possible change in) your work affected your well-being?
  - How does it manifest?
  - How do you cope with it?
  - Why is it better or worse now?

**Coping**

- What kept/keeps you going?
- Why do some people cope better than others? What contributes to this?
- Who or what gives you support?

**Support**

- What do you need regarding to (emotional support)?
- Do you receive support (if you need it)?
  - What did that support look like and how did you find it?
- Positive or negative experiences?
- Comparison over time?
- How would you like the support to look?
  - What would you advise others regarding support?

**The following themes were explored in the survey. Based on the answers, respondents can be asked to explain further:**

- Misunderstanding by others
  - How do you notice this? (example)
  - How does that affect you?
- Appreciation
  - How do you notice this? (example)
  - From whom?
  - How does that affect you?

- Solidarity

| **Characteristics of interview respondents** | | | | | | | |
| --- | --- | --- | --- | --- | --- | --- | --- |
| **Rsp** | **Gender** | **Age** | **Profession** | **Setting** | **Interviews**  **T1 T2 T3** | | |
| **1** | Female | ⩽35 years | Nurse | Hospital | X |  |  |
| **2** | Female | 46-60 years | Nurse | ICU | X | X | X |
| **3** | Female | 36-45 years | Nurse aid | Nursing home | X | X | X |
| **4** | Female | 46-60 years | Nurse aid | Nursing home | X | X | X |
| **5** | Female | ⩽35 years | Nurse aid | Nursing home | X | X | X |
| **6** | Female | 46-60 years | Nurse | Nursing home | X | X |  |
| **7** | Female | 46-60 years | Nurse | Nursing home | X | X | X |
| **8** | Female | ⩽35 years | Nurse | Hospital | X | X | X |
| **9** | Female | 46-60 years | Nurse | Home care & nursing home | X | X | X |
| **10** | Female | 46-60 years | Nurse | Hospice facility | X | X | X |
| **11** | Female | >60 years | Nurse | Home care & hospice facility | X | X | X |
| **12** | Female | 46-60 years | Nurse | Home care & hospice facility | X | X | X |
| **13** | Female | ⩽35 years | Nurse aid | Nursing home | X | X | X |
| **14** | Female | 46-60 years | Nurse | Hospital | X |  |  |
| **15** | Female | ⩽35 years | Nurse | ICU |  | X | X |
| **16** | Female | 46-60 years | Nurse | ICU |  | X | X |
| **17** | Female | ⩽35 years | Nurse | ICU |  | X | X |

Appendix 3. Characteristics of interview respondents
