# Supplementary material for: (Mis)alignment of employer support and needs of healthcare providers in end-of-life care during a healthcare crisis: a longitudinal mixed-method study during the COVID-19 pandemic (the CO-LIVE study)
Source: Palliat Care Soc Pract. 2025 Oct 8;19:26323524241308268. doi: 10.1177/26323524241308268 (PMC12511713; doi:10.1177/26323524241308268)
Supplement: sj-docx-3-pcr-10.1177_26323524241308268 – Supplemental material for (Mis)alignment of employer support and needs of healthcare providers in end-of-life care during a healthcare crisis: a longitudinal mixed-method study during the COVID-19 pandemic (the CO-LIVE study) [file sj-docx-3-pcr-10.1177_26323524241308268.docx]

| **Appendix 1: Original (translated) and recoded questions in the questionnaire** | | |
| --- | --- | --- |
| **Variable** | **Original question** | **Recoded** |
| **Gender** | **What is your gender?**   - Male - Female - Other - I would rather not say |  |
| **Age** | **What is your age?** | **Categorized**  *⩽35 years*  *36-45 years*  *46-60 years*  *>60 years* |
| **Profession** | **In what role were you caring for patients?**  Nursing assistant  Nurse  Physician assistant  General practitioner  Elderly care physician  Other medical specialty, namely:  ___________________________________  Other care professional, namely:  __________________________________  Volunteer | **Categorized**   - Nurse   *Nurse aides, nurse, physician assistant*   - Physician  *General practitioner, elderly care physician, physician with a different specialism* - Other  *Other healthcare professional, volunteer* |
| **Setting** | **In which setting did you provide care?**  *(more than one answer possible)*  At home / community  In a hospital  At an ICU  At a ward for Corona patients (no ICU)  At another ward  In a care home / nursing home  At a ward for Corona patients  At another ward  In a hospice facility (not specifically for Corona patients)  Other (please specify): _______________________________ | **Categorized**   - Home   *At home/community*   - Hospital   *In a hospital (ICU/at ward for COVID patients, at another ward)*   - In a care home / nursing home   *(At a ward for COVID patients, at another ward)*   - Hospice facility   *For COVID patients, not for COVID patients*   - Other   *Other*   - More than one |
| **Needed emotional support** | How much emotional support did you need during [*earlier defined period]*?  ☐ More than usual  As much as usual  ☐ Less than usual |  |
| **Got enough emotional support** | Please rate to what extent you agree with the follow statements when you think about how you felt during [*earlier defined period]*?  I received sufficient emotional support.  Strongly disagree  Disagree  Neutral  Agree   Strongly agree  Don’t know | **Categorized**   - Disagree   *Strongly disagree, disagree*   - Neutral *Neutral* - Agree   *Agree, strongly agree* |
| **Received emotional support from** | From whom did you receive emotional support during [*earlier defined period]*? (more than one answer possible)  ☐ I did not receive emotional support  ☐ From my partner/friends/family  ☐ From my colleagues  ☐ From my employer  ☐ From a professional  ☐ Other, namely (please specify) ____________________________ |  |
| **Wanted emotional support from** | From whom did you want to receive emotional support during [*earlier defined period]*? (more than one answer possible)  ☐ From no one  ☐ From my partner/friends/family  ☐ From my colleagues  ☐ From my employer  ☐ From a professional  ☐ Other, namely (please specify) ____________________________ |  |
